# Comparison of Two Intravenous Propofol Doses after Jugular Administration for Short Non-Surgical Procedures in Red-Eared Sliders (*Trachemys scripta elegans*)

**DOI:** 10.3390/ani14131847

**Published:** 2024-06-21

**Authors:** Lucia Victoria Bel, Paolo Selleri, Carmen Maria Turcu, Constantin Cerbu, Ioana Adriana Matei, Marco Masi, Iulia Melega

**Affiliations:** 1 Department of Surgery and ICU, Faculty of Veterinary Medicine, University of Agricultural Sciences and Veterinary Medicine Cluj-Napoca, Calea Manastur No. 3-5, 400372 Cluj-Napoca, Romania; smaria-carmen.turcu@usamvcluj.ro; 2 Clinica per Animali Esotici, Veterinary Specialists Centre, 00154 Rome, Italy; paolsell@gmail.com (P.S.); marco8912@hotmail.it (M.M.); 3 Department of Infectious Diseases, Faculty of Veterinary Medicine, University of Agricultural Sciences and Veterinary Medicine Cluj-Napoca, Calea Manastur No. 3-5, 400372 Cluj-Napoca, Romania; contantin.cerbu@usamvcluj.ro; 4 Department of Microbiology, Immunology and Epidemiology, Faculty of Veterinary Medicine, University of Agricultural Sciences and Veterinary Medicine Cluj-Napoca, Calea Manastur No. 3-5, 400372 Cluj-Napoca, Romania; ioana.matei@usamvcluj.ro

**Keywords:** propofol, anesthesia, *Trachemys scripta elegans*, turtle, jugular vein

## Abstract

**Simple Summary:**

Anesthesia in red-eared sliders is necessary both for surgical procedures and for imaging techniques. Propofol is a nonbarbiturate anesthetic agent used for induction in many species and should be administered intravenously. Jugular intravenous cannulas provide safe access with the least lymph contamination for anesthetic administration. In this study, red-eared sliders were anesthetized with 5 mg/kg and 10 mg/kg propofol administered in the jugular veins. Our results indicate that the 10 mg/kg dose is efficient for inducing anesthesia for short non-painful procedures, whereas the 5 mg/kg dose did not prove to be enough for anesthetic induction.

**Abstract:**

This study compares the effects of two different doses of propofol administered intravenously (IV), in the jugular vein, to red-eared sliders (*Trachemys scripta elegans*). In this crossover study, 5 or 10 mg/kg propofol was administered to six *Trachemys scripta elegans* after cannulation of the jugular vein. Each turtle received each dose, G1 (5 mg/kg IV) and G2 (10 mg/kg IV), after a 7-day washout period. The parameters evaluated were heart rate, palpebral reflex, cloacal reflex, muscle relaxation, ease of handling, sensitivity to anterior and posterior pinch stimuli, and possibility of intubation. Additionally, respiratory rate was measured when possible, and the times from propofol administration to full recovery and from intubation to extubation were recorded. None of the turtles in G1 could be intubated, and this dose provided little relaxation and ease of handling, with a duration of effect until full recovery of 12.16 ± 8.32 (SD) min for this group. In G2, five out of the six turtles could be intubated, and the duration of effect was 32.33 ± 5.85 (SD) min. Heart rates were influenced by manipulation for catheter placement. There were statistically significant differences (*p* value ≤ 0.05) between the two groups in muscle relaxation degree, handling, cloacal reflex, and possibility of intubation. The 5 mg/kg propofol dose was not sufficient to induce anesthesia, even when administered in the jugular vein, in red-eared sliders. A dose of 10 mg/kg IV or higher should be used.

## 1. Introduction

In reptiles, the onset of anesthesia from inhalatory anesthetics delivered by mask can be prolonged and, in some cases, like turtles, very difficult to achieve. This is due to their anatomical and physiological characteristics, most importantly, the dive reflex. Moreover, freshwater turtles, such as *Trachemys* spp., exhibit large cardiac shunts, owing to the incomplete anatomical division of the ventricle [1]. Due to these shunts, systemic venous blood recirculates into systemic arteries and lowers arterial oxygen partial pressure, slowing the uptake of inhaled anesthesia [2,3,4]. As a consequence, there is a clinical demand for viable injectable anesthetics in turtles.

Propofol (2,6-diisopropylphenol) is a nonbarbiturate anesthetic, used extensively for anesthetic induction in amphibians and reptiles [5,6], that is eliminated through hepatic metabolism to glucuronide metabolites and renal excretion [7]. It provides rapid induction and has been used in lizards [8], snakes [9], and chelonians [10,11,12]. The intramuscular (IM) administration of propofol is not recommended, both because it has been shown to cause inflammation and necrosis in different species [13] and because it is ineffective for producing sedation or anesthesia. Several studies report the use of propofol in reptiles, with a rapid induction time and short recovery, with dosages ranging from 5 to 20 mg/kg [6,8,10,14,15,16,17,18]. Some studies even advise the possibility of intracoelomic [19] or intraosseous [20] administration in red-eared sliders. Nevertheless, the recommended route of administration for propofol in all species is intravenous [15]. We hypothesized that the administered effective dose could be lower due to jugular vein administration, and thus decided on the two doses, 5 and 10 mg/kg.

Venous access is very important for many medical-based procedures, specifically medication administration. It can be challenging in many reptilian species, but mostly in Chelonians, due to the inability to visualize veins and the difficulty in restraint and handling. This is primarily because they can retract their head and limbs into their shell. The most used venipuncture sites in turtles are the subcarapaceal or supravertebral vessel, the dorsal coccygeal vein, the brachial vein, the occipital plexus, and the external jugular vein [15]. The last is the blood sampling site of choice in turtles and tortoises and should be used when possible [21,22]. This recommendation is based on the fact that it carries the least risk of lymph contamination compared to the subcarapaceal plexus or occipital sinus.

In this context, the aim of this study was to compare the sedative and muscle relaxant effects of two dose rates of propofol (5 and 10 mg/kg) in red-eared sliders, administered intravenously via the jugular vein. To our knowledge, this is the first study assessing the effects of propofol administered via this route in *Trachemys scripta elegans*.

## 2. Materials and Methods

### 2.1. Turtles

Six healthy adult female red-eared sliders (*Trachemys scripta elegans*) weighing 1.37 ± 0.593 (SD) kg were used in this study. All turtles were considered clinically healthy based on physical examination and history.

Turtles were anesthetized to perform CT scans with or without a contrast substance, as a method of screening for reproductive disorders. They were privately owned animals, living outside in a pond, from a single owner, who had signed their informed consent. This study was performed in compliance with directive 2010/63/EU as well as the applicable items of the REFLECT statement.

Prior to this study, the animals were acclimatized one day before and were kept in the same environment. Their housing consisted of plastic water tubs, equipped with an area for basking under artificial lighting and an area for retreat, with the temperature of the water and basking spot being in the POTZ—preferred optimum temperature zone—of the species (20 °C to 25 °C in the water and 25 °C to 45 °C for basking). The turtles were provided with UV B lighting and fed a commercial diet with extra green, s every second day. For the purpose of this study and to limit possible complications, all turtles were kept for 10 days and then returned to the owner’s pond.

### 2.2. Study Design

An uncontrolled crossover design was used, meaning that each individual received both dosages, 5 mg/kg (G1) propofol (PropoVet Multidose, 10 mg/mL, Zoetis, Copenhagen, Denmark) and 10 mg/kg propofol (G2), IV, in one of the jugular veins, with a 7-day washout period. On each of the days of the experiments, the turtles were weighed, and an IV cannula was inserted in either the right or the left jugular vein, without any prior sedation. After local antisepsia with chlorhexidine and alcohol, one examiner would insert either a 24 G or a 26 G IV catheter, depending on the size of the animal.

The heads of the turtles were slightly directed ventrally, and the jugular vein was identified. This vessel lies very superficially, running from the tympanic membrane at the jaw angle to the base of the neck, and compression at the level of the coelomic inlet is useful for better visualization. Once the cannulation was successful, 1 mL/kg IV sterile water was used to flush and confirm the correct placement, and propofol was administered in a bolus, in about 10 s. Turtles were then placed back in ventral recumbency, and various measurements were taken every 5 min (heart rate, palpebral reflex, cloacal reflex, muscle relaxation, ease of handling, sensitivity to anterior and posterior pinch stimuli, possibility of intubation). Respiratory rate was measured when possible; when animals were ventilated, this was performed with room air using a small Ambu bag, at 8 breaths/minute. Heart rate (HR) was obtained by Doppler ultrasound (Ultrasound Blood Flow Detector MD4, Sonomed, Warsaw, Poland) after placing the probe at the left thoracic inlet and directing it towards the heart. Palpebral and cloacal reflexes were evaluated by touching the lateral and medial canthus of the eye with a cotton-tipped applicator and the cloacal opening, respectively, and were assigned a score of ‘0’ when absent and ‘1’ when present.

Using the three-point scale of Santos et al. [12], muscle relaxation, ease of handling and sensitivity to pinch stimuli were evaluated. When the turtle kept its head up and retracted, muscle relaxation score was considered to be ‘0’; when the head, legs, and tail retained a mild degree of muscle tone, the muscle relaxation score was considered to be ‘1’; and when the head, tail, and legs remained extended, the muscle relaxation score was ‘2’. In the case of handling, the same principles were applied: ‘0’ when there was difficulty in flexing and extending the head, legs, and tail and in opening the mouth manually; ‘1’ when there was mild resistance to manipulation of the head, legs, and tail, or to opening the animal’s mouth; and ‘2’ when no resistance to manipulation of the head, legs, and tail, or to opening the animal’s mouth, was identified. The score for sensitivity to toe pinch stimulus was considered to be ‘0’ when the withdrawal response after a pinch using tongue forceps performed on both anterior and posterior limbs was accompanied by movement of the head or another limb, and ‘1’ when the absence of a response to the toe pinch or responses suggestive of a spinal reflex (limb withdrawal unaccompanied by limb or head movement) was seen. Intubation was graded ‘0’ when possible and ‘1’ when impossible.

The induction of anesthesia was defined as the time point at which turtles achieved both maximal muscle relaxation and ease of handling. ‘Recovery time’ was considered to be when there was a full return of baseline scores for palpebral reflex, cloacal reflex, muscle relaxation, and ease of handling. The times from propofol administration and full recovery and the time from intubation to extubation were also recorded. To ensure blinding and eliminate assessment bias, assessors were not informed beforehand about the dose administered to the turtles.

The statistical analysis was performed in EpiInfoTM7 (CDC). The means and standard deviation of heart rate were calculated and compared between the two dosage groups using both an ANOVA Parametric Test for Inequality of Population Means and a Kruskal–Wallis test for two groups. For the other variables (muscle relaxation, handling, and sensitivity to pinching degrees), intubation grading and cloacal and palpebral reflex presence frequencies and 95% confidence intervals were calculated, and frequencies were compared using chi-square, and a *p* value < 0.05 was considered to be significant.

## 3. Results

**Figure 1:**
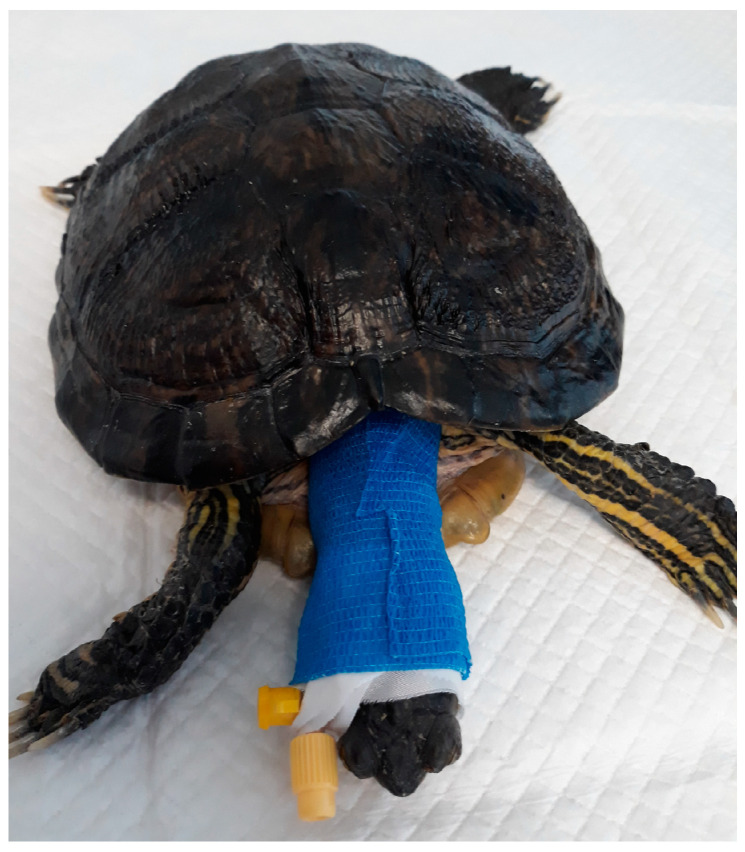
Right jugular vein cannulation with a 24 G IV catheter in a female turtle.

No complications were observed throughout the study and all the blind jugular cannulations were successful (Figure 1). The CT images were unremarkable, showing follicles in different stages of development.

**Figure 2:**
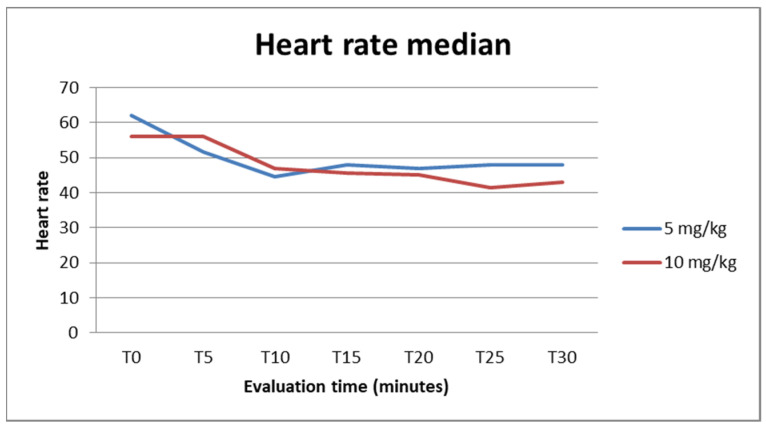
Evolution of median heart rate for both G1 and G2 during the 30-minute evaluation time.

The median heart rate was 48 (25–75% range: 45–60) for G1 and 46 (25–75% range: 42–60) for G2, considering all times. The median for each time measurement can be seen in Figure 2. A lower heart rate mean was observed at times T10 for the group receiving a 5 mg dosage and at T25 for the group receiving 10 mg/kg. When compared, the differences between the means were not statistically significant (*p* > 0.05) whether considering measurements at all times or for each time measurement.

**Figure 3:**
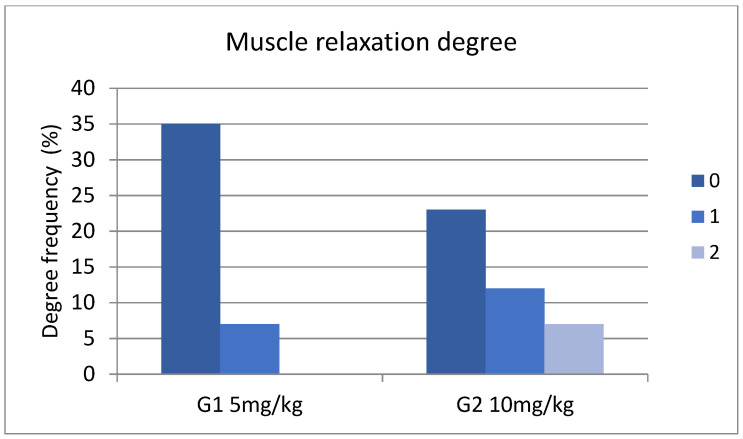
Muscle relaxation degree for G1 (**left**) and G2 (**right**) during the evaluation of the protocol, where 0 = no muscle relaxation, 1 = mild muscle relaxation, and 2 = fully relaxed.

In the case of muscle relaxation degree (0, 1, 2), considering measurements at all times, among the two groups, we observed a statistically significant difference (x^2^ = 10.7985, df = 2, *p* = 0.0045) (Figure 3). Between T20 and T30, the prevalence of degrees of muscle relaxation was similar among the two groups (Supplementary File S1).

**Figure 4:**
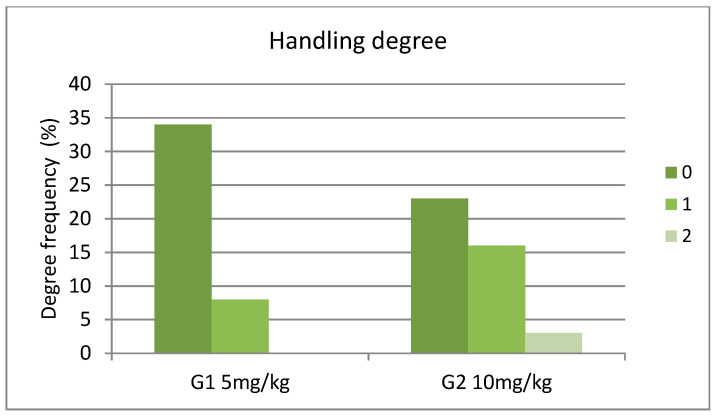
Handling degree for G1 (**left**) and G2 (**right**) during the evaluation of the protocol, where 0 = reactivity to handling, 1 = mild response to handling, and 2 = no response when handled.

Similarly, in the case of handling degree (0, 1, 2), considering measurements at all times, a statistically significant difference was observed among the two groups (x^2^ = 7.7895, df = 2, *p* = 0.0203) (Figure 4). Between T20 and T30 the prevalence of degrees of muscle relaxation was similar among the two groups (Supplementary File S1).

None of the turtles from G1, receiving the 5 mg/kg jugular vein IV propofol dose, could be intubated (Supplementary File S1). On the other hand, when receiving the 10 mg/kg dose, five of the turtles could be intubated (Supplementary File S1) and stayed intubated for 8.6 ± 4.15 (SD) min.

Intubation was possible in four cases (66.67%, 95% CI: 22.28–95.67) at T0 and three cases (50%, 95% CI: 11.81–88.19) at T10 (two of the turtles were still intubated after 10 min), all in the group with 10 mg dosages (Supplementary File S1). The difference in intubation success was statistically significant between the dosage groups when considering measurements at all times (x^2^ = 7.6364, df = 1, *p* = 0.002979).

The cloacal reflex was lost in four cases (66.67%, 95% CI: 22.28–95.67) at T0 and 1 case (16.67%, 95% CI: 0.42–64.12) at both T5 and T10, both in group G2 (Supplementary File S1), with a significant difference between the groups (x^2^ = 6.4615, df = 1, *p* = 0.00645267).

The palpebral reflex was lost in one case (16.67%, 95% CI: 0.42–64.12) at T0 in the group with 10 mg/kg dosages (Supplementary File S1). The difference was not statistically significant between the dosage groups when taking into consideration measurements at all times (x^2^ = 1.012, df = 1, *p* = 0.25).

The duration from T0 (administration of propofol) to intubation was 3.2 ± 2.68 (SD) min for G2, and the duration of effect until full recovery was 12.16 ± 8.32 (SD) min for G1 and 32.33 ± 5.85 (SD) for G2.

In our study, we were not able to evaluate respiration rate throughout the different recorded times, but we could observe apnea in all individuals that could be intubated. Nevertheless, once signs of spontaneous breathing could be observed, extubation followed. We then observed an increase in the respiratory rate, which was also observed in the G2 group, probably because of manipulation for evaluation of the different parameters.

## 4. Discussion

Reptiles, and turtles, in particular, possess a renal portal system. This is a ring of vessels around the kidney, including the cranial portal vein and caudal portal vein, with blood flowing from the caudal area through the coccygeal and iliac veins, that then continues into the afferent renal portal vein, which transfers blood to the kidneys [23,24]. Blood perfusion tubules then leave the kidney through the efferent portal vein, which joins the post-cava vein. This system seems to be involved in kidney perfusion during dehydration, avoiding ischemic damage by ensuring adequate perfusion [25,26]. A system of valves located between the abdominal and the femoral veins either directs the blood flow, influenced by hydration status, directly to the kidneys or bypasses them and directs the flow directly to the circulatory system. This is why the common recommendation is to administer drugs in the cranial part of the body, to avoid this renal portal system, even though there is some controversy surrounding this subject. Some studies have shown that the administration of a combination of the same dosage of dexmedetomidine and ketamine in the cranial or caudal quarter of the body showed a strong difference in sedation power [27,28], and on the contrary, some have proven that the clinical relevance of the renal portal system is minimal and might lead to interactions only in dehydrated animals [29,30].

The anesthetic effects of propofol were evaluated in different reptile species. In *Trachemys scripta* spp., Di Giuseppe et al. [31] evaluated the administration of 5 mg/kg of propofol through the occipital venous sinus (cranial part of the body), the subcarapacial venous sinus (cranial part of the body), and the coccygeal vein (caudal part of the body). When administering the substance in the occipital venous sinus, they observed a full anesthetic plain in all turtles, but this was not the case for the other two groups. They hypothesize that in the case of the subcarapaceal plexus, a higher dosage might be required, 10 or 20 mg/kg as shown by Ziolo et al. [11] and that the poor effect of coccygeal administration is due to the renal portal system. Morici et al. [32] evaluated the difference in response when administering another anesthetic, alfaxalone, at two different sites intravenously in the cranial area: the cervical dorsal sinus and the caudal area, through the coccygeal vein. Their results are consistent with other studies regarding the influence of the renal portal system, proving once again that cranial substance administration will result in a better effect at a lower dosage [32].

Our study is the first to evaluate intravenous propofol administration in the jugular vein in this species. We chose 5 and 10 mg/kg dosages, to identify the lowest dosage that could be used to permit endotracheal intubation in this species, and the jugular vein as it is one of the safest sites for intravenous administration. The location of this vein in turtles is superficial under the skin and can be approachable with or without chemical restraint, depending on the cooperation of the patient. The vein runs on both sides of the neck, in a caudodorsal direction from the dorsal aspect of the tympanum to the coelomic cavity. For better visibility, the cervical folds can be tightened, or in some species, an otoscope can be inserted in the mouth of the animal [22]. The vein can be used for phlebocentesis or catheter placement and substance administration. Once a catheter is in place, it can then also be used for anesthesia induction or supplementation. The 5 mg/kg dose did not prove as effective as the 10 mg/kg dose, and even though the jugular vein is in the cranial part of the body and a very safe route for IV administration (since it is not lymph-contaminated and no shunts will be involved in the excretion of the substances administered via this vein), we did not obtain similar results to Di Giuseppe et al. [31] with occipital venous sinus administration. In their case, the mean induction time was within 1.8 ± 30.8 min (with one terrapin that did not lose the mandibular tone) and the mean tracheal tube insertion time was within 3.89 ± 2.45 min [31]. We obtained a very high standard deviation concerning the induction time; thus, we assume that with the 5 mg/kg propofol dose, there is more of an individual response and that is why our study showed a more stable response at a higher dose, that of 10 mg/kg.

Another reason for such different results might be the influence of the health status of the animals or the environmental temperature. In one study evaluating the administration of alfaxalone in *Trachemys scripta* spp. at two different temperatures, colder temperatures were shown to affect anesthesia and time of recovery [33]. The room temperature in our case was 24 °C, turtles were kept at 26 °C for acclimatization, and cloacal temperature was not evaluated since it is not as relevant compared to its relevance in mammals. Di Giuseppe’s turtles were kept at a temperature 1 °C higher compared to 3.2 ± 2.68 min in the current study. Using a 10 mg/kg dose, Ziolo et al. [11] obtained a mean induction time of 1.7 ± 2.4 min, compared to our 3.2 ± 2.68 min, when administering it in the subcarapaceal plexus. We can only assume that the substance distribution in the blood flow is faster when administered in this area.

The choice of the subcarapaceal sinus as a site for substance administration is mostly related to its ease of access in unsedated animals, with minimal manipulation [34], but is not recommended in clinical practice due to reports of accidental submeningeal injection and clinical abnormalities [35,36]. Kristensen et al. [14] reported in a study the use of atropine and propofol at 15 mg/kg, administered in the subcarpaceal sinus, as described by Ziolo and Bertelsen in 2009 [11]. In their study, repeated dosing of propofol was needed for intubation, with the average propofol dose required being 17.8 mg/kg. Moreover, in one of the turtles (out of eight), induction with propofol was unsuccessful. This might be due to the possible extravasation of the substance when this route is used, a complication best described by Rockwell et al. [37], making the use of the jugular or brachial veins the safest route of IV administration for anesthetic substances when intravenous access is needed. One of the few studies to use propofol administration in the jugular vein in other turtles, Cicarelli et al. [38], used a dose ranging from 5 to 8 mg/kg, for intravenous anesthesia in a marine turtle species, *Caretta caretta*. These dosages allowed a sufficient degree of anesthesia for the minimally invasive procedures they performed. Also, the procedures took around 10–15 min. In the same study, bronchoscopy was performed, and during this time, total intravenous anesthesia with propofol was used. Marine turtles have a higher capacity to maintain apnea than semi-aquatic ones, and none of these turtles were intubated. We would assume that if intubation was required, the propofol dose should have been higher. At the same time, these turtles were a very different species to the semiaquatic one in our study and were not clinically healthy, since all the procedures were performed for diagnostic and treatment purposes for pulmonary disease, hence the lower dose necessary for induction in these individuals.

Concerning the effects of IV propofol on the cardiovascular system, previously, a stable level and even a decrease in HR have been reported in most reptiles [8,11,12,39,40,41] except in the case of Bertelsen et al. [39], which is consistent with our study and those on dogs and cats [42,43,44]. In most individuals, we observed an increase in heart rate, independent of the propofol dose that was used, after administration, even though our T0 was considered to be the moment of propofol administration. We believe that this is due to stress caused by manipulation and cannot be compared to a ‘baseline’ value which would have also been very high, which is consistent with the proof of tachycardia as a consequence of physical restraint [45,46]. Also, respiratory depression has been recorded in various species as a concern when using propofol, like fish [47], amphibians [48,49], reptiles [10], avian [50], and mammalian [51] species. In our case, we observed apnea in all individuals that could be intubated.

Prolonged recovery is a common post-anesthetic complication cited by veterinarians practicing reptile medicine [52]. Compared to other studies [11], we consider the average half-hour recovery time in our study to be efficient for nonsurgical procedures, such as radiology, ultrasound, and CT scanning. The use of other anesthetic substances, like inhalatory anesthesia, sedatives, or opioids, when performing anesthesia for surgical procedures might prolong recovery time, and more research is advised to evaluate more complex anesthetic protocols useful for more complex procedures in red-eared sliders. As there are no analgesic properties to propofol, we recommend pairing it with proven analgesic drugs in turtles undergoing invasive or potentially painful procedures. We would also recommend a thorough pre-anesthetic physical examination and, if needed, at least a protein analysis. There have also been reports of death [22] at high propofol dosages, because propofol is highly protein-bound in vivo, meaning that hypoproteinemia will result in a higher unbound fraction and thus effective dose, possibly leading to an overdose. What our study showed was that propofol administered IV in the jugular vein at 10 mg/kg may be used in red-eared sliders to produce a suitable degree of muscle relaxation for diagnostic or small medical procedures and was enough to intubate five out of six turtles. We have previously used the same dose in practice but injected it IV in the coccygeal vein (personal communication), but this was not enough for intubation. Jugular IV canulation, despite being 100% successful in our case, might be challenging in unsedated turtles, so it could be of future interest to evaluate the use of sedatives like benzodiazepines before propofol administration [53].

**Limitations of the Study**

1. Absence of Control Group: This study lacks a control group. This absence is justified by the lack of a consensus regarding a safe anesthetic protocol in turtles, making it difficult, if not impossible, to establish a positive control group.
2. Small Sample Size: This study includes a small number of turtles due to the limited availability of individuals. As a result, the experiment was conducted as a small-scale crossover trial to limit the variability in the within-sliders comparisons.
3. Generalization Limitation: Because of the small sample size and the absence of a control group, the results of this study cannot be generalized widely. However, they are considered an important advancement toward establishing a specific propofol dose for general use in turtle anesthesia.

These points suggest that while this study has limitations, it represents progress in the field and lays the groundwork for future research.

## 5. Conclusions

The results of this small-scale study show that propofol at 10 mg/kg administered intravenously in the jugular vein provides a fast onset of anesthesia and a short recovery time. Jugular vein cannulation, although slightly challenging, is the safest method of propofol administration, and it should be used whenever possible. The 5 mg/kg IV dose has little effectiveness, but it might be of interest to re-evaluate it in conjunction with the administration of sedatives.

## Data Availability

The data presented in this study are available on request from the corresponding author. The data are not publicly available due to the General Data Protection Regulation.

## Supplementary Materials

The following supporting information can be downloaded at: https://www.mdpi.com/article/10.3390/ani14131847/s1. The data that support the findings of this study are available from the corresponding author, L.V.B., upon reasonable request.
