# Supplementary material for: Comparison of Two Intravenous Propofol Doses after Jugular Administration for Short Non-Surgical Procedures in Red-Eared Sliders (Trachemys scripta elegans)
Source: Animals (Basel). 2024 Jun 21;14(13):1847. doi: 10.3390/ani14131847 (PMC11240516; doi:10.3390/ani14131847)
Supplement: Supplementary file 1 [file animals-14-01847-s001.zip › animals-3012654-supplementary.pdf]

Patient	Doze	Time	HR	MR	H	SP	I	PR	CR
A	5	T0	44	1	1	0	0	0	0
A	5	T5	30	0	1	0	0	0	0
A	5	T10	45	0	0	0	0	0	0
A	5	T15	48	0	0	0	0	0	0
A	5	T20	45	0	0	0	0	0	0
A	5	T25	48	0	0	0	0	0	0
A	5	T30	38	0	0	0	0	0	0
A	10	T0	64	2	1	1	0	0	0
A	10	T5	66	1	1	0	0	0	0
A	10	T10	40	0	1	0	0	0	0
A	10	T15	44	0	1	0	0	0	0
A	10	T20	36	0	0	0	0	0	0
A	10	T25	38	0	0	0	0	0	0
A	10	T30	36	0	0	0	0	0	0
B	5	T0	64	1	1	0	0	0	0
B	5	T5	72	0	0	0	0	0	0
B	5	T10	69	0	0	0	0	0	0
B	5	T15	70	0	0	0	0	0	0
B	5	T20	48	0	0	0	0	0	0
B	5	T25	45	0	0	0	0	0	0
B	5	T30	48	0	0	0	0	0	0
B	10	T0	72	2	2	2	0	0	1
B	10	T5	60	2	2	1	1	0	1
B	10	T10	64	1	1	1	0	0	1
B	10	T15	56	1	1	0	0	0	0
B	10	T20	56	0	1	0	0	0	0
B	10	T25	55	0	0	0	0	0	0
B	10	T30	56	0	0	0	0	0	0
C	5	T0	60	1	1	0	0	0	0
C	5	T5	72	0	0	0	0	0	0
C	5	T10	44	0	0	0	0	0	0
C	5	T15	48	0	0	0	0	0	0
C	5	T20	70	0	0	0	0	0	0
C	5	T25	48	0	0	0	0	0	0
C	5	T30	45	0	0	0	0	0	0
C	10	T0	68	2	1	1	1	0	1
C	10	T5	64	1	1	0	1	0	0
C	10	T10	64	0	0	0	0	0	0
C	10	T15	68	0	0	0	0	0	0
C	10	T20	76	0	0	0	0	0	0
C	10	T25	56	0	0	0	0	0	0
C	10	T30	60	0	0	0	0	0	0
D	5	T0	72	1	1	0	0	0	0
D	5	T5	52	1	1	0	0	0	0
D	5	T10	40	0	0	0	0	0	0

D	5	T15	38	0	0	0	0	0	0
D	5	T20	48	0	0	0	0	0	0
D	5	T25	70	0	0	0	0	0	0
D	5	T30	48	0	0	0	0	0	0
D	10	T0	42	2	1	1	1	0	1
D	10	T5	45	1	1	0	0	0	0
D	10	T10	48	1	0	0	0	0	0
D	10	T15	42	1	0	0	0	0	0
D	10	T20	44	1	0	0	0	0	0
D	10	T25	39	0	0	0	0	0	0
D	10	T30	32	0	0	0	0	0	0
E	5	T0	64	1	1	0	0	0	0
E	5	T5	51	0	0	0	0	0	0
E	5	T10	45	0	0	0	0	0	0
E	5	T15	48	0	0	0	0	0	0
E	5	T20	38	0	0	0	0	0	0
E	5	T25	48	0	0	0	0	0	0
E	5	T30	70	0	0	0	0	0	0
E	10	T0	36	2	1	1	1	1	1
E	10	T5	48	1	1	0	1	0	0
E	10	T10	39	1	1	0	0	0	0
E	10	T15	45	0	0	0	0	0	0
E	10	T20	42	0	0	0	0	0	0
E	10	T25	41	0	0	0	0	0	0
E	10	T30	42	0	0	0	0	0	0
F	5	T0	52	1	1	0	0	0	0
F	5	T5	46	0	0	0	0	0	0
F	5	T10	38	0	0	0	0	0	0
F	5	T15	44	0	0	0	0	0	0
F	5	T20	46	0	0	0	0	0	0
F	5	T25	54	0	0	0	0	0	0
F	5	T30	52	0	0	0	0	0	0
F	10	T0	48	2	2	1	1	0	0
F	10	T5	52	1	1	1	0	0	0
F	10	T10	46	1	1	0	0	0	0
F	10	T15	46	0	0	0	0	0	0
F	10	T20	46	0	0	0	0	0	0
F	10	T25	42	0	0	0	0	0	0
F	10	T30	44	0	0	0	0	0	0

MR= muscle relaxation; degrees 0-2; H= Handling; degrees 0-2; SP = Sensitivity to pinching; degrees 0-2; I= intubation; 0- not possible; 1- possible; PR = palp. Reflex; 0- present; 1- not present; CR = cloacal reflex; 0- present; 1- not present
